# Digital Hologram Watermarking Based on Multiple Deep Neural Networks Training Reconstruction and Attack

**DOI:** 10.3390/s21154977

**Published:** 2021-07-22

**Authors:** Ji-Won Kang, Jae-Eun Lee, Jang-Hwan Choi, Woosuk Kim, Jin-Kyum Kim, Dong-Wook Kim, Young-Ho Seo

**Affiliations:** 1 Department of Electronic Materials Engeering, Kwangwoon University, Kwangwoon-ro 20, Nowon-gu, Seoul 01897, Korea; jwkang@kw.ac.kr (J.-W.K.); wkdghks1206@kw.ac.kr (J.-H.C.); kws@kw.ac.kr (W.K.); jkkim@kw.ac.kr (J.-K.K.); dwkim@kw.ac.kr (D.-W.K.); 2 OLED Team Associate, Siliconworks, Baumoe-ro, Seocho-gu, Seoul 06763, Korea; jaeeunlee@siliconworks.co.kr

**Keywords:** digital hologram, digital watermark, deep neural network (DNN), training dataset, convolution neural network (CNN)

## Abstract

This paper proposes a method to embed and extract a watermark on a digital hologram using a deep neural network. The entire algorithm for watermarking digital holograms consists of three sub-networks. For the robustness of watermarking, an attack simulation is inserted inside the deep neural network. By including attack simulation and holographic reconstruction in the network, the deep neural network for watermarking can simultaneously train invisibility and robustness. We propose a network training method using hologram and reconstruction. After training the proposed network, we analyze the robustness of each attack and perform re-training according to this result to propose a method to improve the robustness. We quantitatively evaluate the results of robustness against various attacks and show the reliability of the proposed technique.

## 1. Introduction

The hologram is a record of a fringe pattern that occurs due to interference between a reference wave as a reference and an object wave reflected on an object and contains two-dimensional (D) data of a complex plane (real and imaginary numbers, or size and phase) but contains 3D image information. The digital hologram may be generated by obtaining an analog hologram in which the interference phenomenon is realized by an optical device with a digital photographing device or sampling the analog hologram to generate a digital hologram. However, in recent years, digital holograms are generated by mathematically modeling and calculating the interference between two waves and by generating them using deep learning [1,2,3].

Recently, digital holograms have been widely used due to the digital hologram compression standard of JPEG Pleno [4], the development of hologram printers [5], and the advent of various holographic displays [6]. With the development of such digital hologram contents, interest in digital hologram security is also increasing. Algorithm-based methods have been studied so far for watermarking digital holograms. Javidi et al. published a study to increase security by generating a 3D object host image and a watermark as a hologram, embedding the watermark on the host image, and performing dual-phase encoding [7]. Kim and Lee presented a study to find the optimal watermark embed strength to minimize MSE (mean square error) between the reconstruction image and the original image [8]. Our research team has also proposed several techniques for holographic watermarking so far. In the first method, a hologram is transformed into a frequency domain using a discrete wavelet transform with a Mallat-tree subband structure. A bit plane of a specific subband is replaced with watermark data [9]. In the second method, a block-based discrete cosine transform was applied to the hologram, and the edge map was extracted to find the position to embed the watermark [10]. Recently, we presented a method of making a hologram into a 2D diffraction pattern using the characteristic of Fresnel transform and embedding a watermark using Fresnel transform and convergence of traveling waves [11]. In another recent study, Zhou et al. proposed a method to embed a watermark by finding a spatiotemporal edge by using both spatial and temporal continuity as a target for holographic video [12].

Artificial intelligence technology using deep learning has been widely used in all fields. Similarly, in the area of digital watermarking, studies using deep learning are increasing. There is no published technology for digital holograms yet, but deep learning-based technologies show good performance in the digital watermarking of 2D images. Zhu et al. proposed a method to embed the watermark globally by duplicating 1D watermark data in 3D [13]. This method consists of an encoder network that embeds a watermark, a decoder network that extracts a watermark from a watermarked image passed through an attack layer, and an adversarial network for steganographic analysis. Ahmadi et al. proposed a method of converting the original image into the frequency domain and embed watermark data in the frequency domain using the DCT transform network that has already been trained [14]. In this method, circular convolution is performed to distribute the watermark data globally. Kim et al. proposed a method to embed the watermark by enlarging the watermark data to the original image resolution instead of lowering the original image to the resolution of the watermark data [15]. Deeba et al. also proposed a neural network that can embed a watermark. This network does not contain attacks and extracts [16].

We intend to try holographic watermarking using deep learning. We show that the deep neural network can adequately interpret the components of the hologram and prove that the deep neural network can hide the watermark invisibly in the hologram. It is shown that the watermark embedded in the hologram can be very robust by including a malicious attack in the deep neural network’s training process. Since the image quality of hologram reconstruction is essential, we train the invisibility of reconstruction simultaneously. We propose a deep neural network (DNN) that performs watermarking of digital holograms and a training method that considers the characteristics of holograms. In addition, if the network is vulnerable to a specific attack after the train, we propose a method to improve the robustness of the attack through re-training.

This paper is organized as follows. In Section 2, the previous works are explained, and in Section 3, the detailed structure of the proposed network is described. In Section 4, the method of constructing the train and the dataset used for the test and the training method are explained, and the experimental results based on this are analyzed. Section 5 concludes this paper.

## 2. Previos Works

In this section, we explain the previous studies about hologram watermarking and deep learning-based watermarking in depth.

### 2.1. Hologram Watermarking

Javidi et al. proposed a technique to use digital holography to hide a 3D object stored in the form of a digital hologram within another 3D object, which generates the digital hologram of both the hidden 3D object and the host 3D object optically using phase shift interferometery. Then, the hologram of the hidden 3D object is encoded using double phase encoding. The encoded hologram of the hidden 3D object is embedded within the hologram of the host 3D object. The watermarked hologram is double phase encoded again using a different set of random phase codes. The second double phase encoding process provides a higher level of security and will guarantee the whiteness of the transmitted hologram. The watermarked hologram is robust to pixels occlusion in terms of reconstructing different poses of the 3D object due to the whiteness of the final transmitted data. Mathematical analysis and digital experiments showed that the proposed system has a reasonable performance [7]. Kim and Lee derived a formula for the optimum weighting factor with which the watermark was embedded into the digital hologram of a 3-D host object. It was shown that the optimum weighting factor minimized the total mean-square-error (MSE) of the reconstructed host object and the decoded watermark. The optimum condition for watermarking the digital hologram of a 3-D host object is analyzed. The experiment showed that the digital hologram watermarked with the optimum weighting factor produces the least errors in the reconstructed 3-D host object and the decoded watermark even in the presence of an occlusion attack [8].

Our team has developed several hologram watermarking methods. We proposed spatial-domain and a frequency-domain electronic watermarking schemes. The spatial-domain scheme was only to compare the results to the ones from frequency-domain scheme and the frequency-domain scheme used two-dimensional mallat-tree discrete wavelet transform. Both of them showed very high imperceptibility and quite high robustness against the attacks. Especially the MDWT-domain scheme was very high in robustness such that the error ratio at the worst case was only 3%. Thus, we expect that it is used as a good watermarking scheme of digital hologram with high performance. However, the spatial-domain scheme turned out to be useless when data compression process is necessary after watermarking [9]. Our team also proposed a hologram domain and a frequency domain watermarking method. For the frequency domain scheme, we used the DCT (Discrete Cosine Transform) as the transforming technique: the segmented and DCTed data space is the target data space to be watermarked. For both schemes, we tried to find the best watermark positioning and embedding schemes. Between the two watermarking schemes, it was obvious that the frequency domain scheme was much better except some extreme cases such as 15% Gaussian noise addition attack [10]. We proposed the Fresnel transform-based watermarking method. In this method, both of the host hologram and the watermark were diffracted more than once for each. The results were refracted to concentrate the diffracted data into the local regions of the transformed plane so that the regions occupied one of them. From this process we changed and selected the region of the watermark. We experimented our scheme with various test images for various attacks on data-manipulating attack and geometric attack. As the experiments, we considered various attacks for both pixel-value changing attacks and geometric attacks. For all the considered attacks which do not ruin the host data too much to be useful, the extracted watermarks were apparently recognizable even though the PSNR values to the original watermark data seemed quite bad [11].

Zhow et al. proposed a spatiotemporal consistent embedding algorithm for the holographic video watermarking. Imperceptibility requirement in the holographic video watermarking is more challenging compared with static holograms because of the temporal dimension existing in videos. The embedding algorithm should not only consider spatially embedding strength for each frame of the video but also take the temporal dimension into account in order to guarantee the visual quality of the moving object. Before embedding, to defend the imperceptibility of the watermark from the holographic moving object, the embedding parameters were evaluated by the salient object from the interframe and intraframe. To ensure the robustness, 3-D watermark converted data (QR code) were embedded in the cellular automata (CA) domains using 3-D CA filters. The QR codes can be extracted from the watermarked holographic frames, and the final 3-D watermark can be digitally reconstructed with different depths’ cue using the computational integral imaging reconstruction algorithm. The experimental results demonstrated that the proposed method exhibits superior performance compared to several methods in the literature, especially the robustness against additive noise and compression attacks [12].

### 2.2. Deep Learning-Based Watermarking

NN-based watermarking schemes have been proposed [13,14,15,16,17,18,19], and their characteristics are described in this section. Zhu proposed a method named as `HiDDeN’, which consists of WM embedding network, noise layer, WM extracting network, and an adversary network. The adversary network was for the steganographic process, which is an additional function but it is used for watermarking function, too. The loss function of adversary network is an adversarial loss, while the embedding and extraction networks use L2 norm loss. The loss function of the watermarking process is the scaled combination of the three, but adversary network uses only the adversarial loss [13]. Ahmadi et al. proposed a scheme to use the DCT frequency domain by implementing and training a network to perform a DCT separately. The network consists of WM embedding network, attack simulation, and WM extraction network. Before entering the network, the host image is reduced to the resolution of WM and DCTed by a DCT network that has been constructed and trained already [14]. Kim et al. proposed a neural network to perform an invisible robust blind watermarking for digital images. It is a convolutional neural network (CNN)-based scheme that consists of pre-processing networks for both host image and watermark, a watermark embedding network, an attack simulation for training, and a watermark extraction network to extract watermark whenever necessary. It has three peculiarities for the application aspect: the first is the adaptability of the host image resolution. It is to apply the proposed method to any resolution of host image and is performed by composing the network without using any resolution-dependent layer or component. The second peculiarity is the adaptability of the watermark information [15]. Deeba et al. employed DNN models for digital watermarking Technology. Watermarking scheme for deep neural networks is proposed which is the black box in terms of verification. Watermarking is applied to recognize the possession of the copyright of digital contents such as audio, images, and videos. They performed the experiment watermark embedding through DNN, in the watermarking process, training the neural network to be as accurate as possible concerning these randomly chosen layers and neural network formalize the idea of backdooring a neural network with specific properties. Authors ran experiment training without the watermark and with the watermark and it turns out that the accuracy of the neural network is almost the same; it sometimes even increases a bit [16]. Mun et al. proposed a watermarking method with an AE-structured NN, consisting of residual blocks. All residual blocks are composed of a unit that performs ReLU after adding CONV(1 × 1)-ReLU-CONV(3 × 3)-ReLU-CONV(1 × 1) and CONV(1 × 1). For the embedding, the host image is reduced to the WM’s resolution by the AE encoding process. To each of the resulting layers, the WM information is added to form the embedding AE’s encoded data, which is entered into the decoder of the embedding AE. This decoder increases the resolution to that of the host image and reduces the resulting number of images to the original host image’s channels [17]. Zhong et al. proposed a scheme to replace the attack simulation with a Frobenius norm. Each of its embedder and extractor networks consists of four connected function networks; one layer (invariance layer) connects the two networks. Thus, each pair network forms a loss function, and the final cost function for training is constructed with the linear combination of the four by determining the four-loss functions by determining the scaling factors empirically [18]. Liu et al. proposed a two-stage training scheme TSDL (two-stage separable deep learning), in which the entire NN with adversary network is trained without any attack (FEAT, free end-to-end adversary training), at first, then in the next train only the extractor without the adversary network is re-trained (ADOT, noise-aware decoder-only training) by adding the attack simulation. In this scheme, the duplicated binary (here, 1, and −1) to the resolution of the host image is concatenated in each convolution layer in the embedder network except the last two layers performing 3 × 3 convolution and 1 × 1 convolution, in order [19].

## 3. Watermarking Network

This section explains the proposed hologram watermarking network and training methodology.

### 3.1. Structure of Neural Network

**Figure 1:**
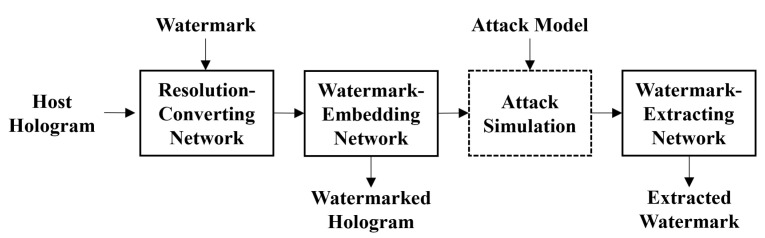
Structure of deep neural network for the proposed watermarking.

The proposed DNN consists of three sub-networks; resolution-converting network (RCN), watermark-embedding network (WMN), and watermark-extracting network (WXN). All networks use 3 × 3 convolutional layer. We show the structure of the DNN for hologram watermarking in Figure 1. The complex data of the host hologram is normalized to the range of [−1, 1]. The watermark has the binary format type of 1 and −1.

**Figure 2:**
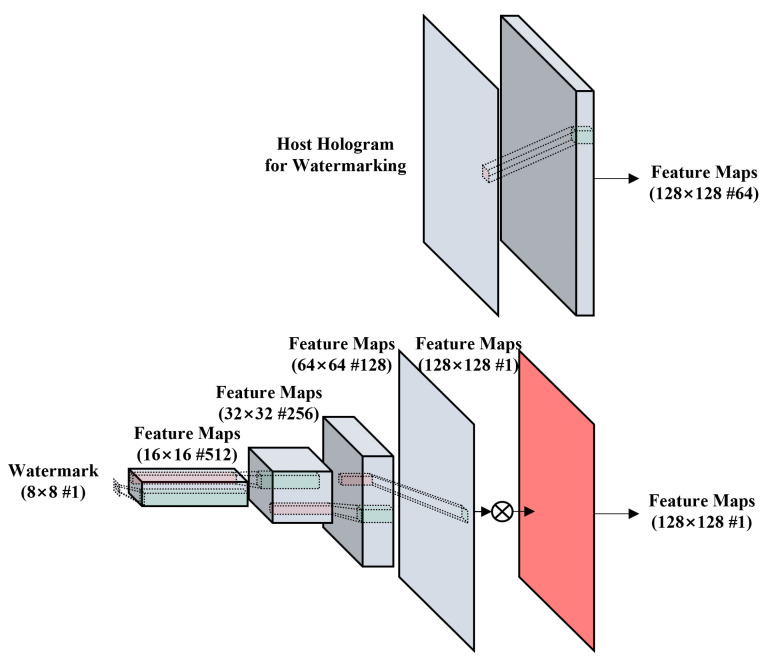
Network structure of the resolution-converting network (RCN).

The RCN consists of the hologram RCN and the watermark RCN. The hologram RCN has a convolution layer that keeps the resolution of channels constant and expands the host hologram to 64 channels. The watermark RCN consists of four convolutional layers, which increases the resolution of a watermark up to the same resolution of the host hologram. The former three layers have the structure of convolution-batch normalization-ReLU(activation)-AP(average pooling), and the last fourth layer has the structure of CONV-AP. After multiplying the strength factor to the output of the last layer, the feature maps are output. The strength factor is decided by experiment. The structure of the RCN is shown in Figure 2.

**Figure 3:**
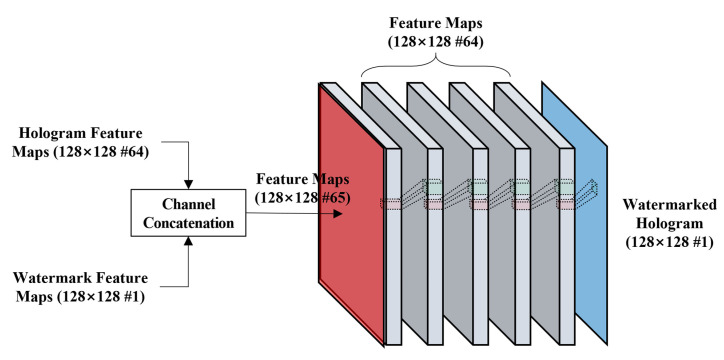
Network structure of the watermark-embedding network (WMN).

The hologram and watermark feature maps with the same resolution are concatenated and formed to a channel in the WMN. The WMN consists of five CNN blocks, which keeps the resolution of the input feature map unchanged. The WMN does not include a pooling layer. The former four layers of the WMN consist of CONV-BN-ReLU, and the last fifth layer consists of CONV-tanh. The reason that the last layer uses the tanh as the activation function is to normalize the output to the range of [−1, 1] to be the same as the host data. The structure of the WMN is shown in Figure 3.

The input of the WXN is the hologram in which the attack was applied to the watermarked hologram. The input hologram is normalized as in the embed process, and watermark information is extracted from the normalized hologram. This result is denormalized again, converted into binary numbers with only −1 and 1 values, and the final watermark is extracted.

**Figure 4:**
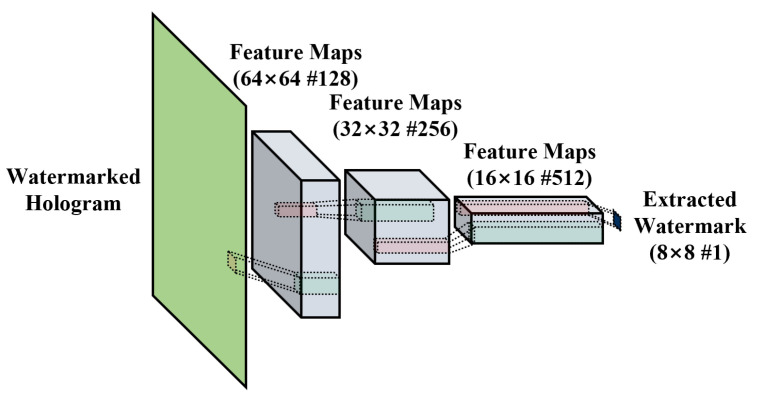
Network structure of the watermark-extracting network (WXN).

The WXN consists of four CNN layers, and the resolution is reduced in each layer so that the output of the last layer has a watermark resolution. The first three layers consist of CONV-BN-ReLU, and the fourth layer uses CONV-tanh so that the output has a binary value of [−1, 1]. If the result range of the fourth layer is [0, 1], 1 is assigned, and if the range is [−1, 0], a value of −1 is assigned. The structure of the WXN is shown in Figure 4.

### 3.2. Training

**Figure 5:**
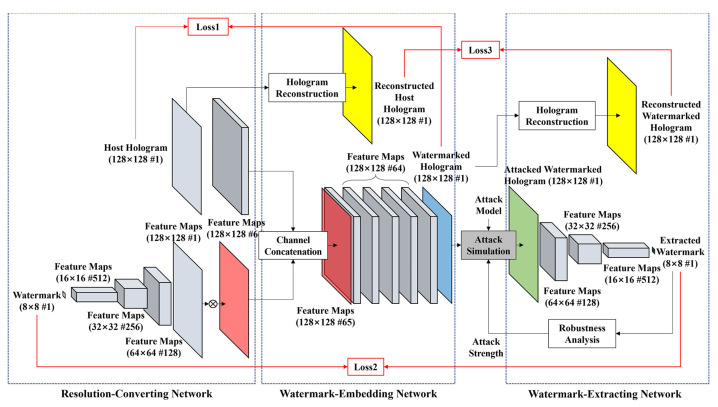
Training methodology of the deep neural network for watermarking.

We propose a training method for hologram watermarking together with the DNN. We try to increase the robustness of hologram watermarking by including attack simulation in the training process. The signal processing-based watermarking algorithm has rules to defend against every predicted attack after analyzing them, but deep learning-based watermarking considers attacks in the training process. The host hologram itself is very important, but the reconstruction of the hologram is more critical for invisibility because users observe not the hologram but the reconstruction. Therefore, we insert the reconstruction of holograms in the training process. Figure 5 shows the proposed training methodology for hologram watermarking. In a 2D or 3D image, the watermark is embedded in the domain that the user observes, but the embedding domain (hologram) is different from the observing domain (reconstruction) in the hologram, so the evaluation of invisibility is conducted in both domains.

As previously explained, the attack for holograms is included in the training process to add robustness to the watermarking algorithm. The reconstruction process is also included because of its importance for invisibility. The pixel-by-pixel correlation between the real and imaginary parts may be expressed as phase information, and the phase information corresponds to the stereoscopic information of a hologram. If the watermarking algorithm does not consider the relationship between real and imaginary parts, it affects the stereoscopic property regardless of the robustness of watermarking. Therefore, the amplitude and phase component should be considered together with the real and imaginary parts to consider the stereoscopic property of the hologram after reconstruction:

- Including attacks for the watermarked holograms.
- Considering reconstruction error of the watermarked holograms.
- Considering stereoscopic quality in reconstruction.

**Figure 6:**
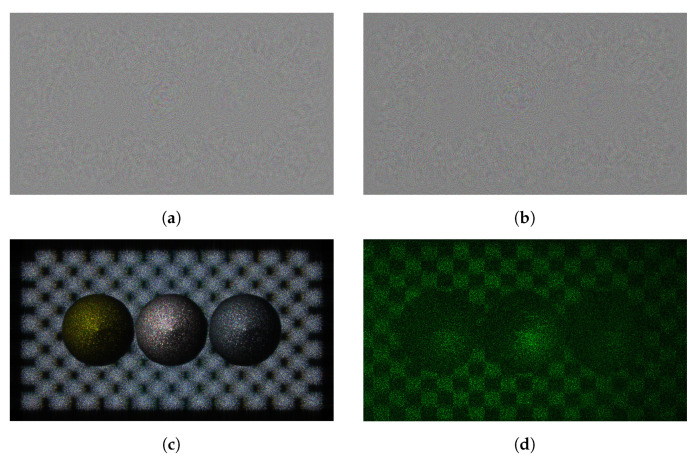
Sphere3 hologram provided by JPEG pleno (**a**) real and (**b**) imaginary hologram, (**c**) ampli-tude of the reconstruction hologram, (**d**) optical reconstruction.

The integrated training method for the DNN is shown in Figure 6. The watermark with a different resolution is converted to the feature map with the same resolution through the RCN. The difference between the original host hologram and the watermarked hologram is used for the first loss function ($L_{1}$). After reconstructing two holograms, the difference between the reconstruction results is used as the second loss function ($L_{2}$). While the DNN is trained with two loss functions, the watermark-embedded hologram and the reconstruction have invisibility against the watermark embedding. Since the visual feature of holograms is like noise, it is very difficult to visually recognize the minute changes of holograms. Next, when the attack is applied to the watermarked hologram, the attacked watermarked hologram is generated. The embedded watermark is extracted from the attacked hologram by the WXN. The difference between the embedded watermark and extracted watermark is used for the third loss function ($L_{3}$).

The first loss function $L_{1}$ is defined as the mean square error (MSE) between the original hologram ($h_{org}$) and the watermarked host ($h_{wmd}$) as described in Equation (1). M × N is the resolution of the host hologram.

(1) $$L_{1}=\frac{1}{MN}\sum_{i=1}^{M}\sum_{j=1}^{N}{\left[h_{org}\left(i,j\right)-h_{wmd}\left(i,j\right)\right]}^{2}$$

The second loss function $L_{2}$ is defined in Equation (2), which is calculated using the mean absolute error (MAE) between the original watermark ($WM_{org}$) and the extracted watermark ($WM_{ext}$).

(2) $$L_{2}=\frac{1}{XY}\sum_{j=1}^{X}\sum_{i=1}^{Y}\left|WM_{org}\left(i,j\right)-WM_{ext}\left(i,j\right)\right|$$

Since the data used in Equation (2) is binary number, we use the MAE in Equation (2) unlike Equation (1). Through experimental result, we identified that the usage of MAE provides better training efficiency and improves the performance of the DNN. Since $L_{3}$ uses the same equation with $L_{1}$; the equation for $L_{1}$ is not defined.

Generally, the invisibility of the embedded watermark has trade-off relationship with robustness. In other words, if a watermark is weakly embedded with small strength factor, invisibility is increased, but robustness is decreased. Conversely, if the watermark is strongly embedded with a large strength factor, invisibility decreases and robustness increases. Since watermarking pursues both invisibility and robustness, two factors cannot be separated. Therefore, in this paper, the loss function $L_{EMB}$ for watermark embedding is defined as in Equation (3), and the loss function $L_{EXT}$ for watermark extract is defined as Equation (4).

(3) $$L_{EMB}=\lambda_{1}L_{1}+\lambda_{2}L_{2}+\lambda_{3}L_{3}$$

(4) $$L_{EXT}=\lambda_{4}L_{2}$$

In Equations (3) and (4), $\lambda_{1}$, $\lambda_{2}$, $\lambda_{3}$, and $\lambda_{4}$ are hyperparameters of the DNN which are decided by experiment.

### 3.3. Re-Training by Robustness Analysis

The proposed DNN can improve its robustness through various training methods. The structure and depth of the network can be variously adjusted, and the dataset for a train can be variously modified. In addition, the form and type of attack can be variously modified, and the performance of the network can be improved by adjusting hyperparameters. We propose a method to improve the robustness by performing re-training for a specific attack after analyzing the robustness against an attack. After training the network, the inference is performed using the trained network. An attack with weak robustness may be selected from the inference result compared to other attacks, or an attack requiring higher robustness may be selected. This process is expressed as robustness analysis in Figure 5. Next, after increasing the frequency of attacks with weak toughness, a re-train is performed. When performing re-training, the number of trains is determined while observing the train results with the desired robustness. Because the tendency of the train depends on the type of attack and the required robustness, the number of trains should be determined experimentally by the researcher.

## 4. Experimental Result

### 4.1. Environment

The proposed DNN was programmed using Python and Tensorflow and implemented in the computing environment of Intel(R) Xeon(R) Gold 5120 CPU @ 2.20 GHz with 180 GB RAM and NVIDIA Tesla V100-SXM2-32GB GPU. A hundred digital hologram patches were used for mini-batch. The training was carried out up to 400 epoch, and the value of 0.0001 was used for the learning rate. For optimization the Adam optimizer ($\beta_{1}=0.5,\beta_{2}=0.999$) was used, and $\lambda_{1}$, $\lambda_{2}$, $\lambda_{3}$, and $\lambda_{4}$ were 45, 0.2, 20, and 45, which were decided by the experiment.

To measure the watermarking performance, we evaluated the invisibility of holograms and reconstruction and the robustness of the extracted watermark. The invisibility was estimated using the peak signal-to-noise ratio (PSNR) of Equation (5), where *m* and *n* are the height and width of a hologram, and $max_{I}$ is the maximum value of a hologram. *i* is a pixel of an original hologram, and $I^{\prime }$ is a pixel of a watermarked hologram. The robustness was assessed using the bit error ratio (BER) which is defined as the number of bit errors. The PSNR of the invisibility was calculated using the original hologram (or the reconstruction) and the watermark-embedded hologram (or the watermark-embedded reconstruction). The BER of the robustness was calculated using the original watermark and the extracted watermark. After dividing holograms into patches of 128 × 128 size, 5000 patches randomly selected were used for the training dataset. Through the same method, 1000 patches for the verification and the inference datasets were selected, respectively. The watermark of 8 × 8 size was used, which has a binary format with only −1 and 1 value.

(5) $$PSNR=10\log_{10}\left(\frac{\max_{I}^{2}}{\frac{1}{mn}\sum_{i=0}^{m-1}\sum_{j=0}^{n-1}{\left|I\left(i,\;j\right)-I^{\prime }\left(i,\;j\right)\right|}^{2}}\right)$$

### 4.2. JPEG Pleno Dataset

JPEG Pleno is an organization that studies the standard compression framework for new image representation formats such as texture-plus-depth, light field, point cloud, and hologram [20]. JPEG pleno constructs and provides a database with various academic institutions and companies [21]. Holograms provided by JPEG Pleno are largely divided into four types: ERC, B-com, UBI, and WUT datasets according to the organization and production technology, and WUT color digital holograms were recently added [22,23,24,25,26,27,28,29]. Table 1 shows the JPEG Pleno dataset. In this paper, we use three sets, excluding the WUT.

Among datasets provided by JPEG pleno, three datasets (ERC, B-com, and UBI) used in this paper include 46 kinds of holograms. We regard the real and imaginary part of the complex hologram with R, G, and B channels as an independent hologram, which means that we can use 172 holograms for our study. Figure 6 shows Sphere3 hologram with the resolution of 1920 × 1080. The pixel pitch for the SLM is 8 μm and the distance of reconstruction is from 0.2783 to 0.2817 m. The hologram has 32-bit floating point format. Figure 6d is the result of optical reconstruction using a spatial light modulator (SLM) with a pixel pitch of 8.0 um. The wavelength is 532 nm.

### 4.3. Training Result

**Figure 7:**
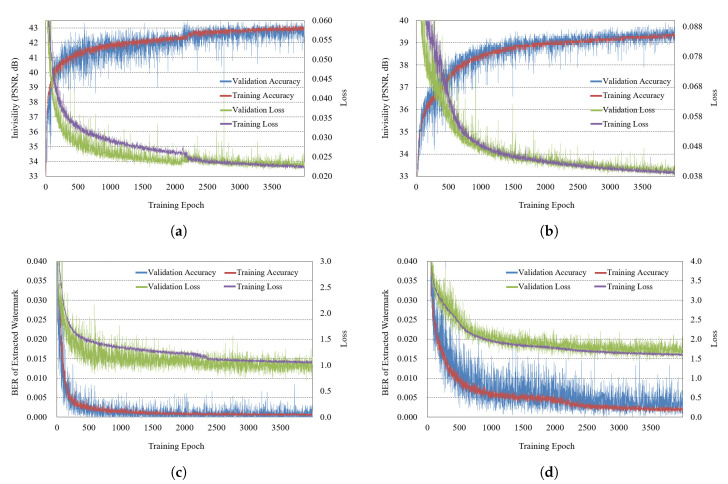
Training result without reconstruction (**a**) watermark embedding (real), (**b**) watermark embedding (amplitude), (**c**) extracting (real), (**d**) extracting (amplitude).

First, we explain the training result of the DNN for watermarking. The training is mainly classified into two cases of reconstruction included and reconstruction not included. Figure 7 shows the training result in which the DNN does not include the reconstruction process. In each subfigure, the blue, red, green, and purple lines correspond to the validation accuracy, training accuracy, validation loss, and training loss, respectively. The performance is converged in about the 4000th epoch. Figure 7a is for embedding a watermark to the real part of a hologram, and Figure 7b is for embedding a watermark to the amplitude part. Figure 7c,d are for the watermark extractions from the real and amplitude parts. The training for extraction in Figure 7c,d includes attack simulation for robustness. In embedding the amplitude part of a hologram, the embedding process affects both the real and imaginary parts, so hologram data changes a lot. In embedding the real part, since the watermark is not embedded in the imaginary part, the PSNR of a hologram may be relatively high. However, it may adversely affect the reconstruction result.

Table 2 shows the results of the invisibility test (inference) performed using the trained watermarking network. In the case of embedding in the real part, the invisibility of the hologram was higher by about 2.2 dB, and the reconstruction result was lower by about 1.5 dB when embedding in the amplitude part. In embedding in the real part, we can predict that the phase correlation of the complex hologram has deteriorated, so it may have affected the reconstruction. Based on the invisibility of the reconstruction result, we found that it is better to train by embedding a watermark in the amplitude part.

**Figure 8:**
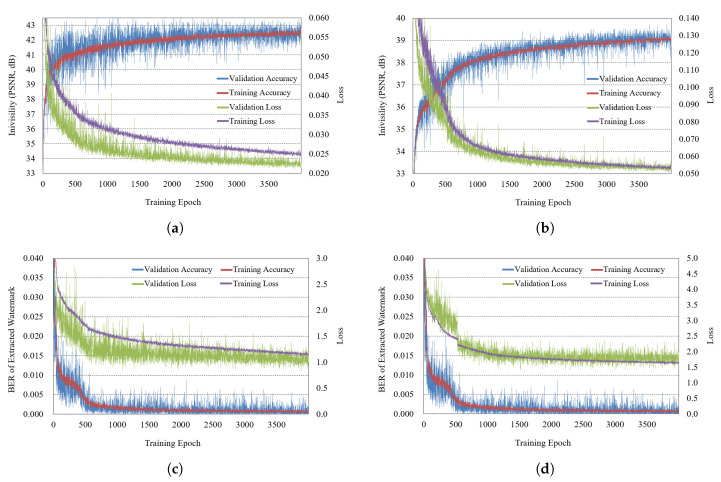
Training result with reconstruction (**a**) watermark embedding (real), (**b**) watermark embedding (amplitude), (**c**) extracting (real), (**d**) extracting (amplitude).

Figure 8 is the result of training with reconstruction added, and it has the same configuration as Figure 7. In Figure 8, the invisibility is somewhat lower in embedding the watermark in the amplitude part than in the case of embedding the watermark in the real part.

In the test (inference) results for invisibility in Table 3, the invisibility of the hologram was 1.0 dB lower in the case of embedding the watermark in the amplitude part than in the case of embedding the watermark in the real part, but the invisibility of the reconstruction was 3.4 dB high. When considering the results of invisibility comprehensively, embedding a watermark in the amplitude part while including the reconstruction process in the train showed the best result at 40.836 dB.

### 4.4. Robustness Result

Table 4 shows the robustness results for the four types of watermarking described above. We included various attacks in the watermarking training process, and most of the results show excellent robustness. We performed a test of 1000 holograms, measured the BER of the extracted watermark, and calculated the average. In Table 4, the last two columns represent the difference in BER results (without reconstruction—with reconstruction) when reconstruction is not included in training and when reconstruction is included. In the average result of the last row, the case where the reconstruction included training showed better results (BER: 7.143, 10.151), and the case where the reconstruction was embedded in the amplitude part showed better results. In the invisibility results, the best results were obtained when reconstruction was included in training and the watermark was embedded in amplitude. In the robustness results, the best results (BER: 7.143) were shown when embedding in the real parts under the same conditions. However, in the case of embedding in amplitude, it is difficult to conclude which one has better performance because the image quality of the reconstruction is 3.4 dB higher.

### 4.5. Re-Training for Robustness

**Figure 9:**
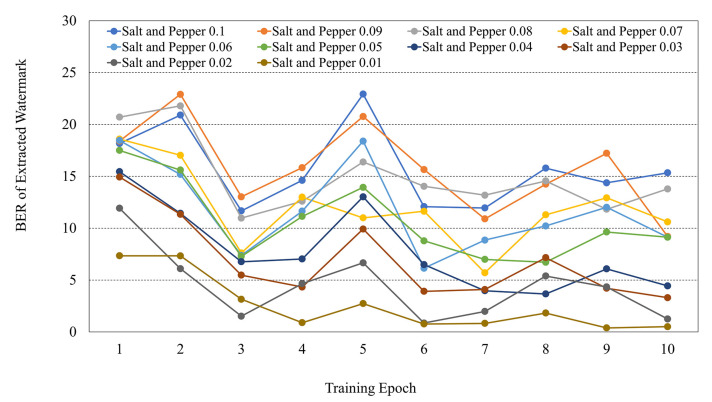
Re-training result for the salt and pepper noise.

From the results of Table 4, the proposed DNN has a good characteristic with a BER of 7∼10% of the extracted watermark while having invisibility of 37∼41 dB. However, some results in Table 4 also show a relatively poor BER of 20∼30%. We proposed a network re-training method to improve such partial low performance. Figure 9 shows the training results for the proposed re-training. In Table 4, when reconstruction is included in training and the watermark is embedded in amplitude, the BER of salt and pepper noise is high. Therefore, we performed 10 epoch re-training for this attack. Although it is possible to obtain a lower BER through more re-training, our purpose is to show that the robustness of the network trained against attacks can be improved through re-training, so only re-training of 10 epochs is performed. The training was conducted by increasing the frequency of training for salt and pepper noise by about 2 times in re-training. In the re-training result of Figure 9, the BER was improved.

Table 5 shows the test results using the weight of the 7th epoch showing the lowest BER among the training of 10 epochs. Through re-training, it was possible to reduce the BER by an average of 62.5%. By combining re-training and various training methods, it is proved that the proposed network can have better robustness.

### 4.6. Visual Analysis

**Figure 10:**
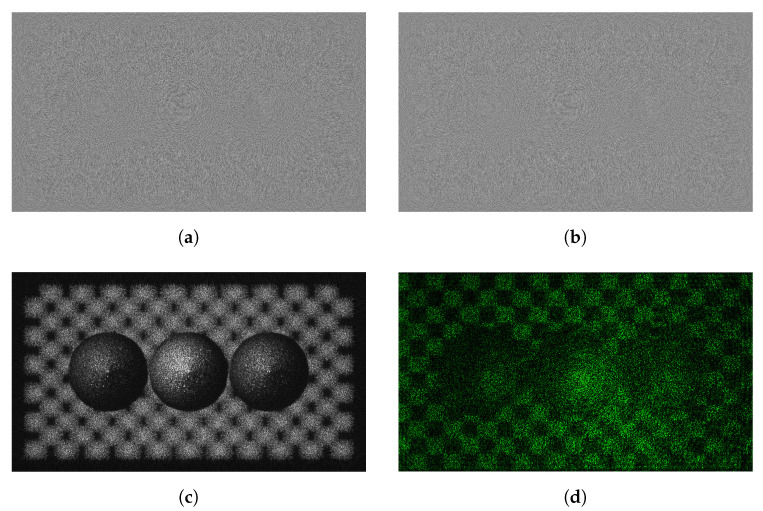
Visual result of the hologram which is watermarked in the real part (**a**) original and (**b**) watermark embedded hologram, (**c**) numerical and (**d**) optical reconstruction result (hologram: 37.829 dB, phase: 26.983 dB, reconstruction: 34.563 dB).

Figure 10 shows the result of embedding the watermark in the real part of the hologram. Figure 10a,b show the real and imaginary parts of the hologram, and the resolution of the hologram used in the experiment is 1920 × 1080. Figure 10c is an image obtained by numerical reconstruction at a distance of 0.2785 m. Figure 10d is the result of optical reconstruction with a pixel pitch of 8.0 um and the wavelength of 532 nm.

**Figure 11:**
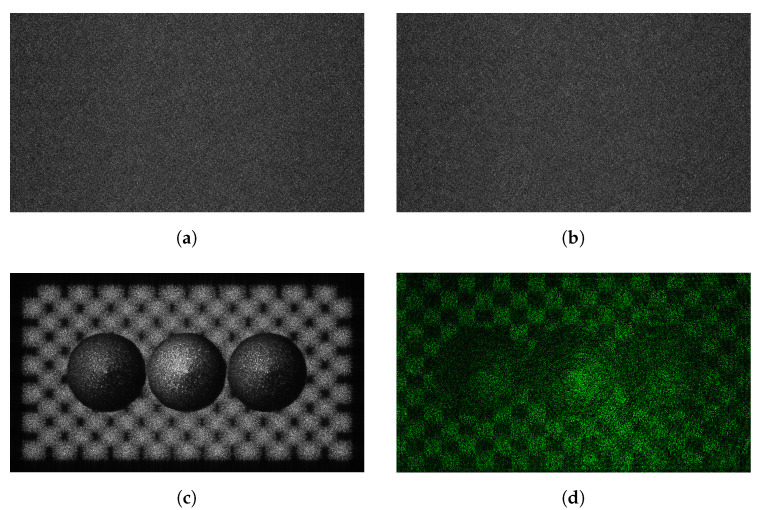
Visual result of the hologram which is watermarked in the amplitude domain (**a**) original and (**b**) watermark embedded hologram, (**c**) numerical and (**d**) optical reconstruction result (hologram: 38.619 dB, phase: 34.874 dB, reconstruction: 41.659 dB).

Figure 11 shows the result of embedding the hologram into the amplitude of the hologram. The experimental conditions are the same as in Figure 10. When comparing Figure 10 and Figure 11 with Figure 1, the numerical and optical reconstruction results show high invisibility for watermarking embedding.

**Figure 12:**
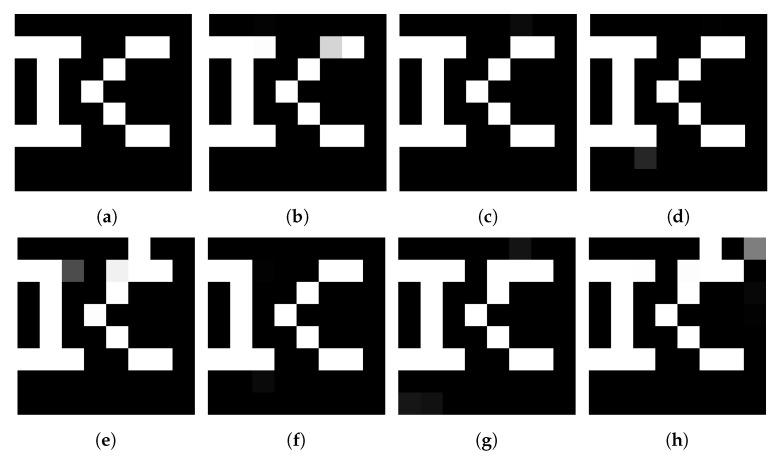
Extracted watermark for various attacks (**a**) original watermark, (**b**) Gaussian blur 3.0, (**c**) averaging (3 × 3), (**d**) sharpening c = 9, (**e**) salt and pepper noise 0.02, (**f**) median filtering (3 × 3), (**g**) JPEG coding (40), (**h**) grid cropping (0.8).

Figure 12 shows the extracted watermarks for several attacks. Although the watermark has a binary format, when it is input to the network and extracted, it is input as 8 bits, so gray components appear in the extracted image. However, numerical measurements are performed using BER. For most attacks, the original information of the extracted watermark can be visually confirmed.

## 5. Conclusions

This paper showed that watermarks could be successfully embedded and extracted in digital holograms using deep learning. For digital holograms, the quality of the reconstructed image is essential, and the three-dimensional effect of the hologram is essential. We included the image quality of reconstruction in training to improve invisibility in the reconstruction of digital holograms. In 2D or 3D images, the quality of the domain itself in which the watermark is embedded is essential. Since the user observes the information reconstructed through the interference and diffraction of the hologram, not the hologram itself, we proposed a training model for the deep neural network by reflecting the unique characteristics of the hologram. We conducted training for both the case where the reconstruction domain was not included in the training and the case where the reconstruction domain was included. As a result, it was confirmed that the robustness was higher when the reconstruction domain was included. In addition, according to the hologram format, it was confirmed that embedding in the amplitude component showed higher robustness and invisibility than embedding in the complex part. According to the result of analyzing the robustness of the attack, it was shown that performance could be improved by re-training the weak attack. Therefore, it was shown that the proposed method can have high versatility for watermarking and it has adaptive characteristics against various attacks.

## Figures and Tables

**Table 1:** Structure of the JPEG Pleno Dataset.

Class	Hologram	Number
ERC 6	Interfere - I [22]	5
	Interfere - II [23]	12
	Interfere - III [24]	7
	Interfere - IV [25]	8
B-com	[26,27]	32
UBI	EmergImg-HoloGrail-v1 [28]	4
	EmergImg-HoloGrail-v2 [28]	6
WUT	[29]	2

**Table 2:** Invisibility of the hologram and reconstruction without reconstruction in training.

Domain	Dataset	PSNR [dB]
Real	Hologram	40.598
	Hologram Phase	12.092
	Reconstruction	38.216
Amplitude	Hologram	38.389
	Hologram Phase	33.733
	Reconstruction	39.731

**Table 3:** Invisibility of the hologram and reconstruction with reconstruction in training.

Domain	Dataset	PSNR [dB]
Real	Hologram	40.125
	Hologram Phase	11.748
	Reconstruction	37.423
Amplitude	Hologram	39.195
	Hologram Phase	33.862
	Reconstruction	40.836

**Table 4:** Robustness result for the extracted watermark (average BER).

Attack	Strength	Without Reconstruction		With Reconstruction		Difference	
		Real	Amplitude	Real	Amplitude	Real	Amplitude
Identity		0.929	0.673	0.957	0.088	−0.028	−0.215
Gaussian Blurring	1	2.151	1.359	2.504	1.675	−0.353	−0.316
	2	2.559	6.86	2.535	8.865	0.024	−2.005
	3	18.216	32.35	7.138	33.542	11.078	−1.192
	4	20.484	41.098	32.6	39.918	−12.116	1.18
Average Filtering	5× 5	2.895	5.098	2.711	3.539	0.184	1.559
	3 × 3	2.335	1.265	2.784	1.526	−0.449	−0.261
Median Filtering	3 × 3	0.935	2.007	0.909	2.198	0.026	−0.191
	5 × 5	1.736	4.353	1.233	5.004	0.503	−0.651
	7 × 7	32.932	30.473	15.126	34.457	17.806	−3.984
Salt and Pepper Noise	0.1	3.433	25.425	2.999	20.484	0.434	4.491
	0.08	2.465	22.995	2.259	17.954	0.2066	5.041
	0.06	2.177	20.673	1.723	15.918	0.454	4.755
	0.04	1.977	13.798	1.563	15.278	0.414	−1.48
	0.02	1.293	11.934	1.406	9.603	−0.113	2.331
Gaussian Noise	0.1	13.822	17.059	11.968	13.327	1.854	3.732
	0.08	9.501	13.262	7.391	9.586	2.11	3.676
	0.05	2.784	6.812	1.903	4.334	0.881	2.478
	0.03	1.048	2.478	1.035	1.769	0.013	0.709
	0.01	0.935	0.697	0.964	0.903	−0.029	−0.206
Sharpening	9	2.088	1.306	2.233	1.378	−0.145	−0.072
	5	2.122	1.272	2.257	1.309	−0.135	−0.037
Restoration	1	10.137	8.917	9.616	8.401	0.521	0.516
	2	10.818	8.813	10.085	8.561	0.733	0.252
	3	5.495	5.171	4.878	4.36	0.617	0.811
	4	3.702	3.305	3.57	3.407	0.132	−0.102
	5	2.53	2.372	3.175	2.448	−0.645	−0.076
	6	2.166	3.639	2.852	4.497	−0.686	−0.858
Crop Out	0.8	27.908	35.052	23.396	35.605	4.512	−0.553
	0.6	19.201	25.23	15.464	24.924	3.737	0.306
	0.4	12.415	17.517	11.243	15.51	1.172	2.007
	0.2	4.681	5.911	5.33	7.641	−0.649	−1.73
Rotation	15	4.499	5.119	4.021	6.313	0.478	−1.194
	30	8.531	8.585	7.402	8.826	1.129	−0.241
	45	9.829	9.905	8.433	12.63	1.396	−2.725
	60	8.776	9.572	8.073	9.62	0.703	−0.048
	75	5.516	5.532	3.707	4.01	1.809	1.522
	90	0.929	0.673	0.957	0.888	−0.028	−0.215
JPEG	10	46.558	26.597	37.337	32.077	9.221	−5.48
	20	37.35	31.105	33.125	31.031	4.225	0.074
	30	17.732	28.974	27.886	7.181	−10.154	21.793
	40	2.862	26.903	6.033	6.046	−3.171	20.857
	50	2.207	4.319	2.552	2.669	−0.345	1.65
	60	1.827	4.473	2.062	2.257	−0.235	2.216
	70	1.528	0.972	1.623	1.94	−0.095	−0.968
	80	1.198	2.27	1.322	0.97	−0.124	1.3
	90	1.05	0.684	1.044	0.914	0.006	−0.23
	100	1.376	0.76	1.489	1.048	−0.113	−0.288
Average		7.909	11.367	7.143	10.151	0.766	1.216

**Table 5:** Robustness comparison after re-training the proposed network for the attack (salt and pepper noise) with weak robustness.

Attack	Strength	Re-Training		Reduced Rate
		Before	After 7 Epoch	
Salt and Pepper Noise	0.1	20.484	11.947	41.676%
	0.09	24.262	10.894	55.099%
	0.08	17.954	13.175	26.618%
	0.07	22.211	5.701	74.333%
	0.06	15.918	8.848	44.415%
	0.05	18.414	6.980	62.094%
	0.04	15.278	3.967	74.035%
	0.03	17.281	4.084	76.367%
	0.02	9.603	1.968	79.506%
	0.01	9.004	0.816	90.937%
	Average			62.508%
